# A machine learning based depression screening framework using temporal domain features of the electroencephalography signals

**DOI:** 10.1371/journal.pone.0299127

**Published:** 2024-03-27

**Authors:** Sheharyar Khan, Sanay Muhammad Umar Saeed, Jaroslav Frnda, Aamir Arsalan, Rashid Amin, Rahma Gantassi, Sadam Hussain Noorani

**Affiliations:** 1 Department of Computer Engineering, University of Engineering and Technology Taxila, Taxila, Pakistan; 2 Department of Quantitative Methods and Economic Informatics, Faculty of Operation and Economics of Transport and Communications, University of Zilina, Zilina, Slovakia; 3 Department of Telecommunications, Faculty of Electrical Engineering and Computer Science, VSB Technical University of Ostrava, Ostrava, Czech Republic; 4 Department of Software Engineering, Fatima Jinnah Women University, Rawalpindi, Pakistan; 5 Department of Computer Science, University of Chakwal, Chakwal, Pakistan; 6 Department of Electrical Engineering, Chonnam National University, Gwangju, South Korea; Universiti Tunku Abdul Rahman, MALAYSIA

## Abstract

Depression is a serious mental health disorder affecting millions of individuals worldwide. Timely and precise recognition of depression is vital for appropriate mediation and effective treatment. Electroencephalography (EEG) has surfaced as a promising tool for inspecting the neural correlates of depression and therefore, has the potential to contribute to the diagnosis of depression effectively. This study presents an EEG-based mental depressive disorder detection mechanism using a publicly available EEG dataset called Multi-modal Open Dataset for Mental-disorder Analysis (MODMA). This study uses EEG data acquired from 55 participants using 3 electrodes in the resting-state condition. Twelve temporal domain features are extracted from the EEG data by creating a non-overlapping window of 10 seconds, which is presented to a novel feature selection mechanism. The feature selection algorithm selects the optimum chunk of attributes with the highest discriminative power to classify the mental depressive disorders patients and healthy controls. The selected EEG attributes are classified using three different classification algorithms i.e., Best- First (BF) Tree, k-nearest neighbor (KNN), and AdaBoost. The highest classification accuracy of 96.36% is achieved using BF-Tree using a feature vector length of 12. The proposed mental depressive classification scheme outperforms the existing state-of-the-art depression classification schemes in terms of the number of electrodes used for EEG recording, feature vector length, and the achieved classification accuracy. The proposed framework could be used in psychiatric settings, providing valuable support to psychiatrists.

## 1 Introduction

Depression refers to a complex and multifaceted mental health disorder that can affect human health. Depression arises from a persistent deficiency of hormones in the human brain referred to as “dopamine,” which plays a crucial role in various positive processes within the human brain, including self-balance and motivation. Depression has varying degrees of intensity, ranging from mild to moderate to severe [1]. Depression leads to severe health issues like sleep disorders, chronic fatigue, weakened immune system, weight fluctuations, and digestive problems. Depression is a prevalent state impacting individuals of all age groups, genders, and ethnicity across the globe. Every year, about 13% of children, 46% of teenagers, and 19% of adults worldwide suffer from mental illness [2]. As per the statistics reported by World Health Organization (WHO) in the year 2021, approximately 280 million people worldwide are living with depression, accounting for 4.4% of the world’s community [3, 4]. By 2023, depression the most widely spread disease globally, is propelled to be the second highest contributor to transience and disability.

Emotional, physical, and cognitive well-being is relentlessly influenced by depression [5]. Typical indicators associated with depression include melancholy, diminished enthusiasm or enjoyment, alterations in eating or sleeping behaviors, trouble in focusing, sensations of insignificance or repentance, and contemplations of self-imposed harm or self-annihilation. Furthermore, the social and professional performance of an individual is also substantially impaired by continuous exposure to depressive circumstances [6]. This can lead to decreased time efficiency and increased truancy in the workplace. In addition to these factors, more prolonged exposure to depression can trigger the risk of developing other chronic diseases which can cause economic crises both directly (medical expenses of the treatment) and indirectly (decreased efficacy of an individual). WHO estimated that stress, depression, and anxiety disorders cost $1 trillion yearly to the global economy due to the severe degradation in the productivity of individuals [7].

The primary challenge concerning the recognition of depression lies in the social stigma related to mental health conditions. Subsequently, a substantial number of individuals undergoing depression fail to receive the best possible treatment. Therefore, the probe of suitable and efficient schemes to diagnose depression is an evolving area of research. The latest innovations in instrument or sensor technology present new prospects for diagnosing depression. Depression is frequently accompanied by additional mental health conditions, including anxiety, substance abuse, and eating disorders which can complicate the diagnoses and treatment, hence necessitating a holistic approach to tackle various conditions concurrently.

The identification of depression characteristically depends on clinical evaluation, including interviews and questionnaires governed by healthcare professionals. These questionnaires help mental health specialists gather information about a person’s symptoms, seriousness, and overall functioning, which aids in making a judgment. Some of the commonly employed questionnaires include Patient Health Questionnaire (PHQ-9) [8], Beck Depression Inventory (BDI) [9], Hamilton Rating Scale for Depression (HAM-D) [10], Montgomery-Åsberg Depression Rating Scale (MADRS) [11], and Geriatric Depression Scale (GDS) [12].

There are numerous physiological sensors that have been investigated for the estimation of depression. Some of the common physiological measures employed for the recognition of depression include electroencephalography (EEG) [13], electrocardiography (ECG) [14], heart rate variability (HRV) [15], galvanic skin response (GSR) [16], actigraphy [17], and speech signals [18]. Physiological sensors used for analyzing depression offer several compensations over traditional questionnaires developed by psychologists. In contrast to questionnaires, which stack on subjective self-reporting, physiological sensors can provide more precise and dependable data. Physiological sensors allow for real-time analysis of a person’s physiological situation. This uninterrupted monitoring can capture fluctuations in physiological parameters that may indicate the presence or gravity of depression, even when the subject is not deliberately aware of it.

Questionnaires, on the other hand, provide a snapshot of a person’s self-reported indicators at a specific time instant. Moreover, physiological sensors have the prospective to detect early signs of depression before the warning sign become evident to the individual or are reported via questionnaires. This early detection can lead to timely intermediations and prevent the progression of the condition. Furthermore, questionnaires can be persuaded by various biases and hence are not capable of accurately communicating one’s feelings. Physiological sensors evade these biases by directly measuring objective physiological markers, providing a more robust assessment of a person’s mental state.

EEG signals have several advantages over other physiological indicators when it comes to the detection of depression. Prior research works have indicated that EEG can be employed as an effective instrument in the detection and assessment of depression [19–22]. EEG measures the activity of the brain directly by recording the electrical signals produced by neurons. This makes it an effective instrument for investigating brain function and spotting irregularities coupled with depression. Moreover, EEG has a very high temporal resolution, meaning it can portray variations in brain activity in real-time with millisecond precision [23–25]. This is fundamental for sensing rapid fluctuations and dynamics of brain activity connected to emotional processing, which can be influential in understanding depression. Furthermore, EEG is a non-invasive procedure that implies placing electrodes on the scalp, yielding it an unharmed and affluent method for determining brain activity. It does not involve any surgical procedures or the use of ionizing radiation, contrasting with some other neuroimaging procedures. In terms of cost, EEG apparatus is reasonably economical compared to other neuroimaging equipment such as functional magnetic resonance imaging (fMRI) [26]. This makes it handier and cost-lucrative for large-scale studies and clinical purposes. EEG gadgets can be made compact and wearable, letting measurements to be recorded beside clinical settings, such as in a patient’s home or natural setting. This flexibility supports long-term examination and evaluation of depression symptoms, providing a more thorough estimation of the situation. Over and above that, EEG data has the potential to be used for personalized solutions. EEG signals can be used to recognize distinctive patterns or markers coupled with different subtypes of depression or treatment responses [27]. This can help in adapting treatments to individual patients, leading to more efficient and personalized intrusions.

Our study proposes an EEG-based mechanism for detecting mental depressive disorder, utilizing the publicly available Multi-modal Open Dataset for Mental-disorder Analysis (MODMA). EEG data from 55 participants, collected in the resting-state condition with 3 electrodes, is analyzed. Temporal domain features are extracted through non-overlapping 10-second windows and subjected to a novel feature selection mechanism. The algorithm identifies the most discriminative attributes, crucial for classifying mental depressive disorder patients and healthy controls. Classification is performed using three algorithms: Best-First (BF) Tree, k-nearest neighbor (KNN), and AdaBoost. Remarkably, the BF-Tree achieves the highest accuracy of 96.36%, surpassing existing state-of-the-art schemes in electrode usage, feature vector length, and overall classification accuracy.

## 2 Related work

In the last decade, several techniques have employed EEG signals for the recognition of depression. A review of the studies conducted for depression detection using EEG signals has been presented in [28]. In [1], the authors have conducted a study to classify depression patients and healthy controls using EEG data from three electrodes. The proposed scheme was able to distinguish depressed and healthy individuals with an accuracy of 72.25%. A study focusing on the detection of depression using geometric features of the EEG signals is furnished in [29]. The binary particle swarm optimization algorithm was used for feature selection and an accuracy of 98.79% was achieved using the kNN classifier. A depression detection scheme using a content-based ensemble method (CBEM) for EEG signal and eye movement data is discussed in [30]. The proposed method was evaluated on two different datasets comprising of data for 36 and 34 subjects, with a resulting accuracy of 82.5% and 92.65%, respectively.

A pervasive approach for depression detection using EEG signals is materialized in [31]. The authors used EEG data recorded in the rest-state as well as under sound stimulations recorded via using three electrodes. An accuracy of 79.27% is achieved for discriminating depression patients from healthy subjects using kNN classifiers. Moreover, it was also found that the absolute power of the theta band was the most significant characteristic for detecting depression conditions. Another study to examine the long-lasting effect of depression using EEG signals is put forward in [32]. Linear and non-linear features from the EEG signals were delivered to the ensemble voting classifier which predicted an accuracy of 82.55%. A depression classification mechanism using spatial features from the EEG data in response to a cognitive task consisting of positive and negative emotional facial expressions is described in [33]. The depression classification accuracy of 84% and 85.7% is achieved for positive and negative stimuli, respectively.

A machine learning framework for major depressive disorder using EEG signals is shown in [34]. The authors of the study were able to reach an accuracy of 83.3% for depression classification using the kNN classifier. A deep neural network-based classification framework for classifying depression is portrayed in [35]. An accuracy of 93.5% and 96.0% is achieved using the features from the left and right hemispheres, respectively. A seven-electrode EEG-based study aimed at the detection of depression in response to positive emotion-eliciting images is discussed in [36]. The core idea of the study was to establish the fact that depression is associated with blunted positive emotions and there would be a significant difference between EEG data of healthy and depressed individuals which would help in depression classification. The highest accuracy of 83.64% is achieved using a conformal kernel support vector machine classifier.

A cross-subject depression classification scheme using non-linear features of EEG signals recorded in the eye-open and eye-closed state is presented in [37]. A classification accuracy of 94.03% is achieved by support vector machine classifier using the fusion of features extracted from the EEG recorded in the eye-open and eye-closed state. A spatiotemporal feature extraction approach for diagnosing depression using EEG signals is debated in [38]. Convolutional Neural Network (CNN) and Gated Recurrent Unit (GRU) were engaged to extract the features from the recorded EEG data and an accuracy of 89.63% is attained for the proposed framework. A 1D-CNN and GRU network-based deep learning model has been developed for depression detection using EEG data in a study conducted in [39]. An accuracy of 99.33% and 97.98% is achieved for depression detection on a public and private EEG-based dataset, respectively.

In most of the studies available in the literature, the procedure of gathering EEG data utilizes caps and headsets that had a substantial number of electrodes placed closely together. This crowded arrangement of electrodes leads to a huge computational burden when treating the acquired data, rendering it impractical for everyday use. Most studies cited in available literature take on datasets that were gathered by the researchers themselves and are not open to the public for further research purposes. In this study, we have employed a publicly available EEG-based MODMA (A multi-modal open dataset for mental-disorder analysis) dataset available at http://modma.lzu.edu.cn/data/index/ for our analysis. The MODMA dataset encompasses resting-state EEG data collected from 55 participants (29 healthy and 26 depressed individuals), using three frontal electrodes. This paper aims at the classification of recorded EEG signals for mental depressive disorder patients and healthy controls. Furthermore, the study delves into inspecting the impact of feature selection to improve the capability of the proposed system to discriminate between healthy and depressed individuals. A set of twelve temporal features is derived from the captured EEG data. Subsequently, a correlation-based approach is exercised to opt for the most appropriate subset of features by performing channel and feature selection. To the best of our knowledge, there has not been any EEG-based research that has inspected the influence of temporal domain characteristics on the wearable 3-electrode EEG MODMA dataset. The primary advancements brought about by this research are,

1. A framework to distinguish between individuals who are mentally healthy and those who are experiencing depression by analyzing the temporal domain features of the EEG signals obtained during a resting state is developed.
2. The effects of the feature selection technique specifically designed for human depression detection using EEG signals classification are examined.

The rest of the paper is constituted as follows. Section III explains the important stages of the proposed framework for distinguishing between mentally healthy individuals and those with depression. In Section IV, the experimental consequences and performance evaluations of the proposed method are presented. Section V offers a comparative analysis of the achieved results with the existing cutting-edge approaches found in previous research. Finally, Section VI concludes the paper along with future work.

## 3 Proposed methodology

**Fig 1:**
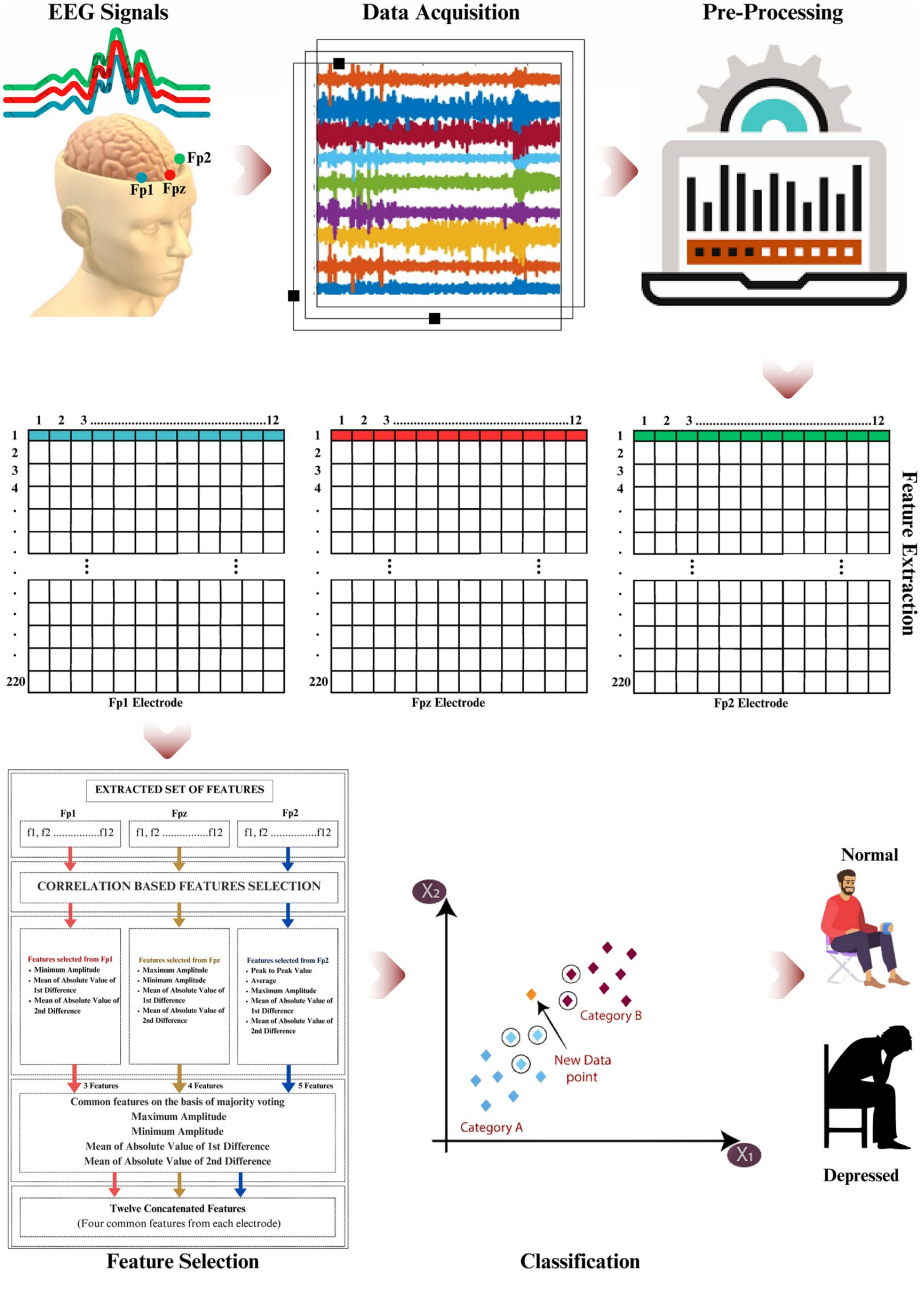
An illustration of the proposed EEG-based mental depressive disorder detection framework.

Fig 1 presents the depression detection framework developed in this study. It encompasses four phases, namely pre-processing, feature extraction, feature selection, and classification. The details regarding each of these blocks are expanded in the sub-sections below.

### 3.1 EEG signal acquisition

The proposed depression classification framework is based on MODMA [40], which is a publicly available EEG dataset. Although we did not collect the EEG data ourselves, the subsequent subsections provide comprehensive explanations of the data acquisition process for the sake of completeness. The choice of dataset for the proposed depression classification scheme is mainly motivated by the existing literature citing the association of frontal area of the brain with the psychological activities like thoughts, emotions, anxiety, stress and depression [41–45] and the idea to keep the number of electrodes to a minimum value, focusing on improvement of the classification accuracy of the existing depression classification schemes. In one of the classical studies regarding functional lobe function and dysfunction, it has been reported that depression is most closely related to frontal lobe of the brain [46]. Another study presented in [47], reported the fact that there exists a relationship between the pre-frontal EEG electrodes and depression diagnoses. Furthermore, a sizable number of studies available in the literature have advocated the fact that frontal EEG electrodes play an important role in diagnoses of mental depressive disorders [48–50]. Therefore, Fp1, Fp2 and Fpz electrode signals have been extensively used for the diagnoses of depression. Fp1 and Fp2 are located on the left and right side of the forehead, respectively where they are positioned between the front and polar regions of the brain. However, FpZ is positioned at the midline of the forehead i.e., it is located between Fp1 and Fp2, representing the central frontal region of the brain.

#### 3.1.1 Participants

A total of 55 participants which included 26 (15 males and 11 females) subjects who have been diagnosed with depression and 29 (19 males and 10 females) subjects who are healthy were recruited for the experiment. Both the depressed and normal participants belonged to the age bracket of 18-53 years. None of the participants were consuming any medicine and were right-handed. None of the individuals participating in the experiment had any medical or neurological disease. Furthermore, the participants were told to abstain from coffee for at least 2 hours and from alcohol for at least 24 hours prior to the experiment. Prior to EEG recording, all participants received detailed information regarding the objectives and protocols of the experiment for data acquisition. Furthermore, all experimental procedures were steered in compliance with pertinent rules and regulations. Written informed consent was obtained from all participants prior to the experiment. The study design were approved by the local Ethics Committee for Biomedical Research at the Lanzhou University Second Hospital in accordance to the Code of Ethics of the World Medical Association (Declaration of Helsinki).

#### 3.1.2 Experimental protocol

**Fig 2:**
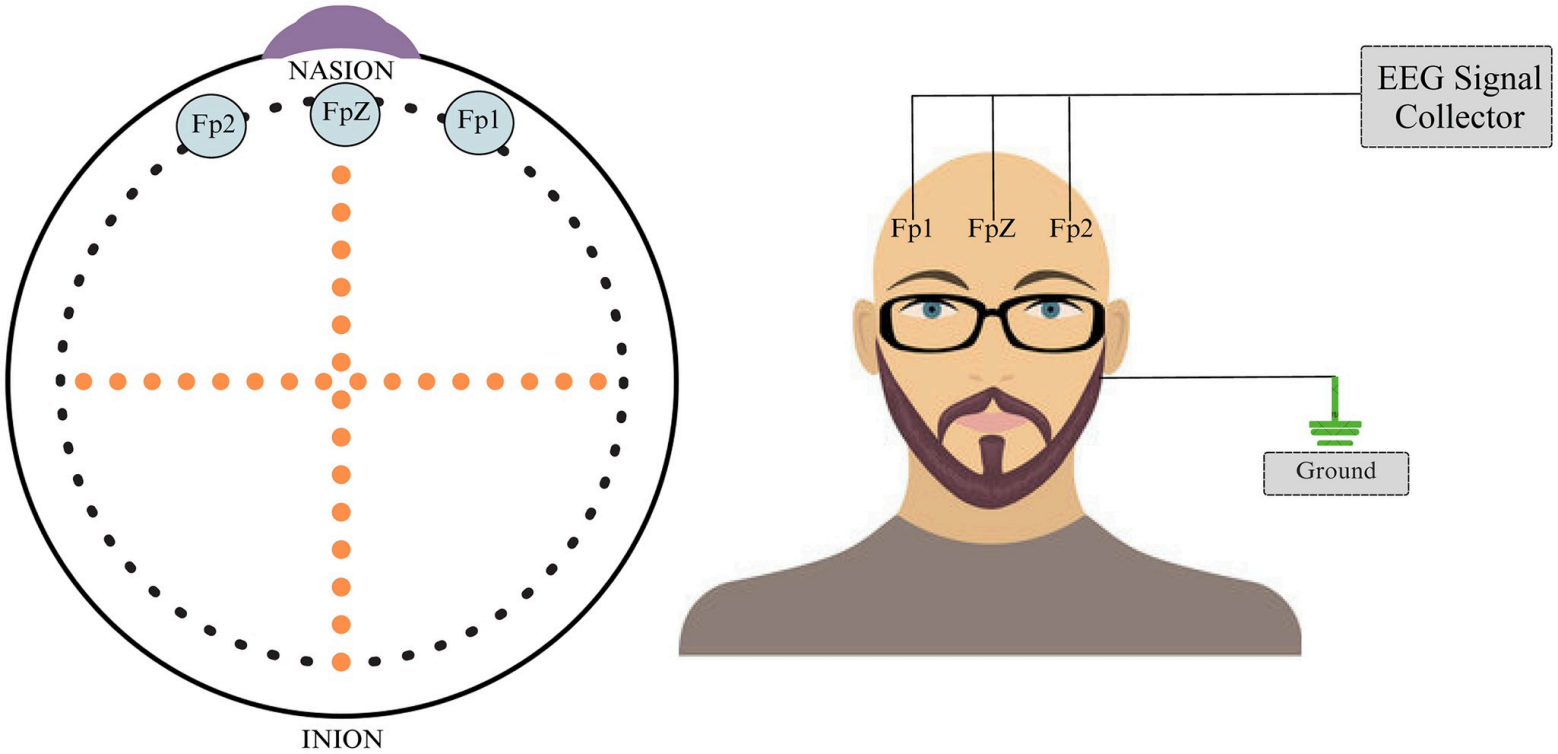
The positioning of the three electrodes and the portable EEG device designed for collecting pervasive EEG signals.

The dataset embraced for the current study consists of clinically diagnosed depression patients and normal healthy individuals. The patients whose EEG data has been acquired have been carefully selected by the professional psychiatrists in the hospital. EEG data of the participants were recorded in rest-state using a custom-designed headset consisting of three electrodes i.e., *Fp*1, *Fpz*, and *Fp*2. Unlike traditional electrodes, the ones used in this study were dry and did not require the application of conductive gel at frequent intervals during data recording. The selection of these electrodes was persuaded by the strong connection of emotional processes to the pre-frontal region of the human brain. Furthermore, these electrodes were positioned in a location on the scalp that was not obstructed by the hair, thus ensuring a high-quality EEG signal. In addition to these three electrodes, a reference is also needed for the optimal recording of the EEG signals. This reference electrode is placed on the mastoid position in this study. The mastoid is a bone positioned behind the ear, and it is part of the temporal lobe. This mastoid bone is frequently employed as a reference point for the EEG electrodes. Electrodes placed on the mastoid aids to establish a reference for recording the brain activity. This reference is required to measure the voltage differences among different brain regions in a precise manner [51]. To curtail the prospective effect of eye movement on the gathered EEG data, the participant’s eyes were deliberately closed right through the recording process. Additionally, a cautious attempt was made to preserve coherent lighting conditions to lower the influence of visual stimuli on brain activity. Fig 2 shows the electrode placement for *Fp*1, *Fpz*, and *Fp*2 according to 10-20 electrode positioning system.

### 3.2 Pre-processing

Pre-processing of EEG signals is a critical stride in investigating and interpreting human brain activity. It implicates a series of techniques to eliminate noise, artifacts, and other unnecessary components from the raw EEG data, allowing for better scrutiny and analysis of the underlying brain signals. It consists of two steps i.e., noise removal and segmentation. Average reference was applied by computing the average of all electrode signals and subtracting it from each electrode’s signal. This average reduced common noise across the electrodes. Moreover, a notch filter of 50 Hz is applied to the recorded EEG signals to remove power-line interference caused by electrical sources. The choice of notch filter of 50 Hz to curtail powerline interference in the recorded EEG signal is originated from the fact that the EEG recording of the MODMA dataset was performed from patients in Lanzhou University Second Hospital, Gansu, China, and the frequency of power lines in China is 50 Hz. A similar technique to remove the power line noise from the MODMA dataset has been employed in depression classification studies in [40, 51, 52]. Furthermore, the EEG data was passed through a third-order smoothing filter to truncate the noisy peaks occurring due to eye blinks and movements, and muscular activity. MODMA dataset consists of 90 seconds of EEG recording from each of the 55 participants. However, in the current study, we have only utilized a 40-second chunk of recorded EEG data. Moreover, we have segmented the data using a 10-second non-overlapping sliding window for the purpose of feature extraction.

The optimal window size for EEG signals in depression classification varies depending on the specific study and the features extracted from the data. In one of the studies available in the literature, a 1.5-second window size was chosen for EEG signals of participants, which provided the best results in terms of classification accuracy for depression classification [53]. Another study investigated the classification of EEG-emotion signals using wavelet entropy and support vector machines, achieving an accuracy of 65% for both valence and arousal using signal segments of 3 to 12 seconds [54]. The mental stress classification scheme presented in [55] reported an optimum window size of 3-12 seconds. A review of studies on depression diagnosis using EEG signals found that various window sizes have been used in studies ranging from 1 to 24 seconds [56]. A window size of 10 seconds has also been reported to achieve good classification accuracy for depression classification in a range of studies [57, 58]. In our proposed depression classification scheme, we experimented with different window sizes ranging from 1-12 seconds with a step size of 1. The best results for classifying the depressed and healthy subjects were achieved using a window segment of 10 seconds which we have reported in the manuscript.

Moreover, the values of 1-12 shown along the columns in pre-processing part of Fig 1 of the manuscript depicts the number of features extracted from each of the Fp1, Fp2, FpZ electrode resulting in a total of 12 x 3 = 36 features. On the other hand, the rows showing value of 1 to 220 depict the number of instances computed for the experiment. The total number of participants in this study is 55 and the EEG data from each participant is comprised of 40 seconds. As mentioned earlier, in this study, we have selected a window size of 10 seconds, thus each participant has 4 chunks of data i.e., 10 seconds x 4 = 40 seconds. Therefore, the number of instances for 55 participants is 55 x 4 = 220 instances constituting a final feature vector of dimension 220 x 36 which is used for feature extraction.

### 3.3 Feature extraction

Next to pre-processing of the EEG data, feature extraction is performed. A set of twelve time domain statistical features are extracted from each channel of the cleaned EEG signal. These features include maximum and minimum amplitude, mean, standard deviation, kurtosis, skewness, peak-to-peak signal value, peak-to-peak time, mean of absolute values of first and second difference, energy, and entropy. Temporal domain features are often simpler and more straightforward to interpret. Temporal domain features are more robust to noise and artifacts present in EEG signals. By focusing on the temporal aspects of the signal, these features may capture relevant patterns while minimizing the impact of irrelevant noise. Calculating temporal domain features is generally computationally more efficient than frequency or wavelet domain analysis. Temporal domain features provide a holistic view of the signal over time, capturing global characteristics. This can be advantageous in scenarios when the goal is to identify overall trends or patterns e.g., depression screening. Some of these features have been employed in studies aimed at the discrimination of healthy and depressive patients using EEG signals [31, 59]. Furthermore, many of these features have been used in a variety of EEG based studies which include motor imagery [60], sleep stage classification [61], eye movement classification [62], and epilepsy detection [63]. The effectiveness of these features has been exhibited in the classification of various states, leading to the conviction that they can also be valuable in the classification of depression.

Details about the extracted set of features and their mathematical representation are produced in the sub-sections below.

#### 3.3.1 Maximum amplitude

The maximum amplitude in an EEG signal corresponds to the highest value attained by the waveform during a specific time interval. The maximum amplitude feature can be an effective measure for characterizing the magnitude of brainwave activity at a certain point in time, and it can provide insights into brain functioning, abnormalities, or specific events of interest in EEG analysis. Mathematically it is represented as,

$$\begin{array}{c} s_{e_{max}}=max\left\{s_{e}\left[n\right]\right\}, \end{array}$$ (1)

where $s_{e_{max}}$ signifies the highest recorded value of the EEG signal *s*_*e*_[*n*] acquired by electrode e. The value of electrode *e* can have a value of *Fp*1, *Fpz*, or *Fp*2.

#### 3.3.2 Minimum amplitude

The minimum amplitude in an EEG signal refers to the minimal value reached by the voltage or amplitude of the recorded brain waves. The minimum amplitude feature can be of interest in several settings, such as recognizing specific patterns or abnormalities in the EEG signal. It can offer insights into the presence of certain brain activities, abnormalities, or changes in brain states. Mathematically minimum amplitude is represented by,

$$\begin{array}{c} s_{e_{min}}=min\left\{s_{e}\left[n\right]\right\}, \end{array}$$ (2)

where $s_{e_{max}}$ signifies the lowest recorded value of the EEG signal *s*_*e*_[*n*] acquired by electrode e.

#### 3.3.3 Mean value

The mean value of an EEG signal is found by computing the average or arithmetic mean of the voltage values over a precise time interval or within a restricted set of data points. It provides a measure of the central tendency of the EEG signal during that phase. In mathematical terms, the mean value is denoted as,

$$\begin{array}{c} s_{e_{mean}}=\frac{1}{N}\sum_{i=1}^{T}s_{e}\left[n\right], \end{array}$$ (3)

where $s_{e_{mean}}$ represents the mean value for EEG data obtained from electrode *e*, and *N* is the total number of samples in a particular time interval.

#### 3.3.4 Standard deviation

The standard deviation is a statistical measure that supplies information about the variability or dispersion of the signal’s amplitude values. It put a figure on how much the data points vary from the mean value. Higher standard deviation values imply larger variability, whereas lower values advocate more coherent or stable brain activity. In mathematical terms, it is expressed as,

$$\begin{array}{c} s_{e_{std}}=\sqrt{\frac{1}{N}\sum_{i=1}^{T}{\left(s_{e}\left[n\right]-s_{e_{mean}}\right)}^{2}}, \end{array}$$ (4)

where $s_{e_{std}}$ represents the standard deviation value for EEG data obtained from electrode *e*.

#### 3.3.5 Kurtosis

Kurtosis is a statistical marker that assesses the shape of a distribution. In the context of EEG signals, kurtosis can be used as a characteristic to illustrate the distribution of amplitudes or frequencies. A positive kurtosis value specifies a distribution with heavier tails and a sharper peak, while a negative kurtosis value signifies a distribution with lighter tails and a flatter peak compared to a normal distribution. EEG signals with high kurtosis may specify the phantom of abnormal brain activity, whilst signals with low kurtosis may denote more standard or Gaussian-like brain activity.

$$\begin{array}{c} s_{e_{K}}=E\left[\frac{\left[{\left(s_{e}\left[n\right]-s_{e_{mean}}\right)}^{4}\right]}{{\left[{\left(s_{e}\left[n\right]-s_{e_{mean}}\right)}^{2}\right]}^{2}}\right], \end{array}$$ (5)

where $s_{e_{K}}$ represents the kurtosis value for EEG data obtained from electrode *e*, and *E* corresponds to the expected value.

#### 3.3.6 Skewness

Skewness is a statistical property that depicts the asymmetry of a probability distribution. From the perspective of EEG signals, skewness can grant information about the shape and symmetry of the signal’s amplitude distribution. Understanding the skewness of EEG signals can offer insights into the underlying neural activity patterns. Positive skewness might reveal the occurrence of surges or high-amplitude events, while negative skewness might imply the domination of low-amplitude activity.

$$\begin{array}{c} s_{e_{skew}}=\frac{1}{N}\sum_{i=1}^{T}{\left[\frac{s_{e}\left[n\right]-s_{e_{mean}}}{s_{e_{std}}}\right]}^{3}, \end{array}$$ (6)

where $s_{e_{skew}}$ represents the skewness of the data from electrode *e*.

#### 3.3.7 Peak to peak signal value

The peak-to-peak signal value is a characteristic frequently used to characterize the amplitude of an EEG signal. The peak-to-peak value is evaluated by finding the absolute difference between the highest and lowest points in a given time window of the EEG signal. This measure presents an indication of the range of amplitudes present in the signal during that time interval. The peak-to-peak signal value can offer an understanding of the overall amplitude of the EEG signal, which can be indicative of the strength of brain activity during specific events or states. In mathematical terms, it can be expressed as.

$$\begin{array}{c} S_{e_{p2p}}=s_{e_{max}}-s_{e_{min}}, \end{array}$$ (7)

where $S_{e_{p2p}}$ is signal peak to peak value.

#### 3.3.8 Peak to peak time

The peak-to-peak time feature of an EEG signal refers to the duration between the positive and negative peaks of a single wave or cycle in the signal. It assesses the time interval from the highest peak to the lowest trough of the waveform. In EEG analysis, peak-to-peak time can provide valued information about the temporal characteristics of the brain activity being recorded. By examining the peak-to-peak time, researchers and clinicians can gain insights into the timing and dynamics of neural activity. A mathematical representation of the peak-to-peak time is given by,

$$\begin{array}{c} t_{e_{p2p}}=t_{e_{pos}}-t_{e_{neg}}, \end{array}$$ (8)

where $t_{e_{p2p}}$ represents the peak-to-peak time, and $t_{e_{pos}}$ and $t_{e_{neg}}$ presents the time at which EEG signal reaches its positive and negative peak, respectively.

#### 3.3.9 Mean of absolute values of first difference

The mean of the absolute values of the first difference feature of an EEG signal refers to a measure of the average magnitude of the changes between sequential samples in the EEG signal. This feature is often used to quantify the variability in the signal. Mathematically it is represented as,

$$\begin{array}{c} MAD_{f_{e}}=\frac{1}{N}\sum \left|s_{e}\left[n+1\right]-s_{e}\left[n\right]\right|, \end{array}$$ (9)

where $MAD_{f_{e}}$ represents the mean of absolute values of first difference.

#### 3.3.10 Mean of absolute values of second difference

The mean of the absolute values of the second difference feature of an EEG signal can provide information about the overall rate of change in the signal. The second difference feature of an EEG signal refers to the discrete second derivative of the signal. Mathematically it is elaborated as,

$$\begin{array}{c} MAD_{s_{e}}=\frac{1}{N}\sum \left|s_{e}\left[n\right]-2*s_{e}\left[n-1\right]+s_{e}\left[n-2\right]\right|, \end{array}$$ (10)

where $MAD_{s_{e}}$ represents the mean of absolute values of second difference.

#### 3.3.11 Energy

The energy of an EEG signal corresponds to the quantity of signal energy present within a specific time interval. Higher energy values imply a larger magnitude signal, which may link to intensified brain activity. The mathematical representation of energy is given by,

$$\begin{array}{c} E_{e}=\frac{1}{N}\sum_{i=1}^{N}s_{e}\left[n\right], \end{array}$$ (11)

#### 3.3.12 Shannon entropy

Shannon entropy estimates the average amount of information or vagueness in a signal. It aims to enumerate the randomness or unpredictability of the EEG signal. Higher entropy values indicate a more complex or irregular signal. Shannon entropy is mathematically explained by,

$$\begin{array}{c} SE_{e}=\sum_{i=1}^{N}P_{i_{e}}\left(log\left(P_{i_{e}}\right)\right), \end{array}$$ (12)

where *SE*_*e*_ is the Shannon entropy for electrode *e*, and $P_{i_{e}}$ represents the probability of the *i*^*th*^ bin of a particular time interval for electrode *e*.

### 3.4 Feature selection

**Fig 3:**
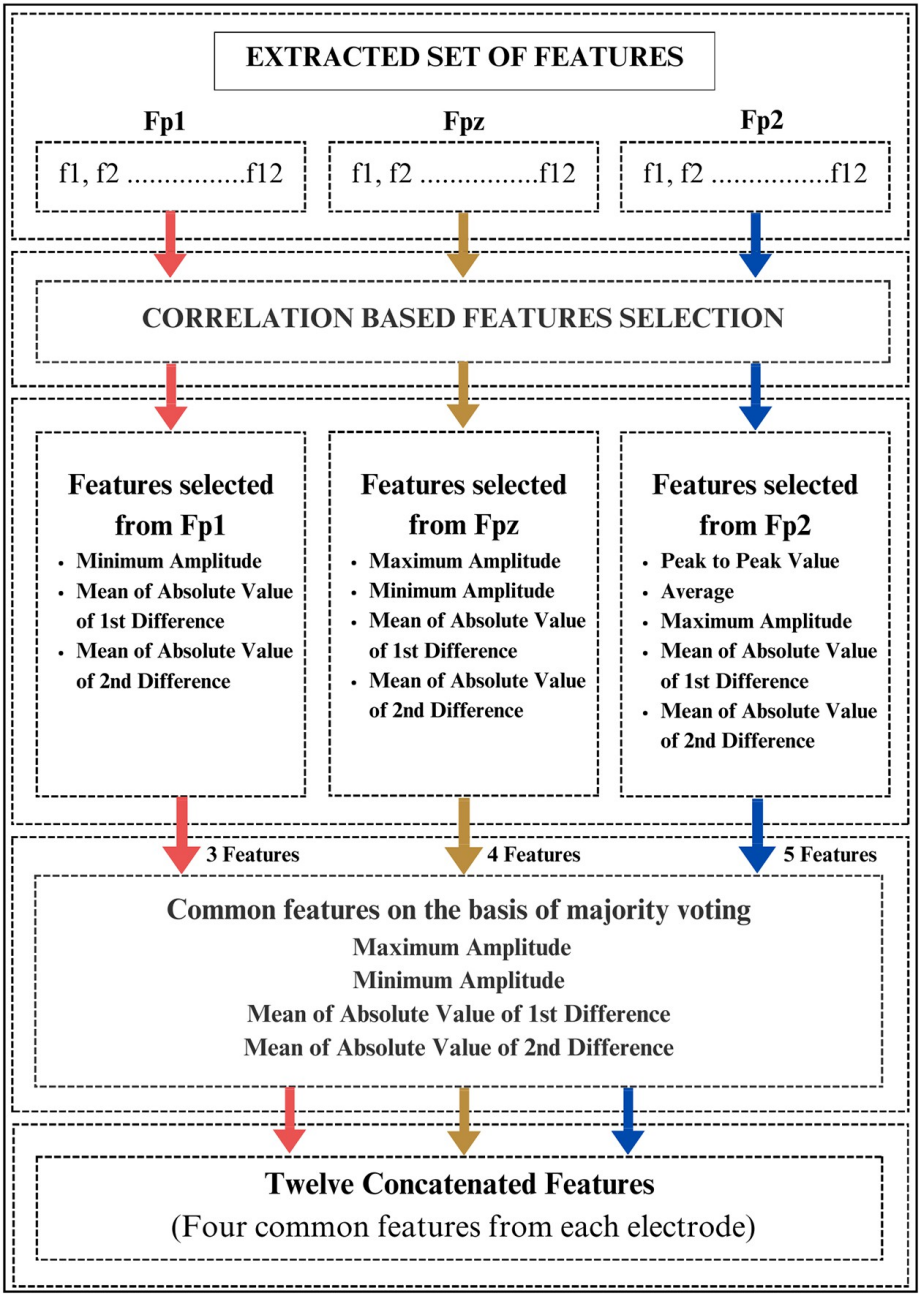
Feature selection scheme for the proposed mental depressive disorder using EEG signals.

The extracted features are concatenated to compose a feature vector which is then subjected to a feature selection process using the correlation-based feature selection (CFS) method. CFS is an algorithm for feature selection to recognize the most significant and useful features for a given problem. CFS aims to find a split of features that are highly associated with the target variable while being minimally correlated with each other. The correlation-based feature selection algorithm aims to achieve an equilibrium between high correlation with the target variable and low redundancy among the selected features. Selecting a subset of relevant features, can enhance model performance, mitigate overfitting, and augment interpretability. In the current study, the feature selection is applied individually to the features extracted from each electrode of the headset and then the common features among the majority of electrodes are selected for the purpose of depressive vs normal subject classification. Fig 3 presents the proposed feature selection methodology for the current study.

The left hemisphere of the brain processes positive emotions, whereas the right hemisphere processes negative emotions. Since depression belongs to negative emotion, therefore more features are contributed Fp2 electrode, which is located on the right hemisphere. Individuals exhibiting greater activity in the right frontal lobe tend to experience more intense negative emotions compared to those with higher activity in the left frontal lobe [64–66]. In a study conducted in [67],it has been reported that alpha asymmetry among individuals with major depression suggests heightened activity in the right parietal lobe compared to the left. Conversely, those with “pure” depression exhibit reduced activity in the right parietal lobe. Therefore, the different feature selection from each of the left and right hemisphere is inline with the finding of the many existing studies in the literature thus supporting our feature selection strategy.

### 3.5 Classification

In our proposed depression detection study, three different classification algorithms are used for classification which includes k-nearest neighbors, AdaBoost, and Best-First (BF) Tree. A concise overview of each of the classifiers is elaborated in the sub-sections below.

#### 3.5.1 K-nearest neighbors

The k-NN algorithm is a straightforward, but helpful supervised machine learning algorithm used for both classification and regression tasks. It is a non-parametric algorithm, meaning it makes no assumptions about the underlying data distribution. The central idea behind the k-NN algorithm is to predict the value of a new data point by glancing at its k-nearest neighbors in the training dataset. The algorithm works out the distances amongst the new data point and all the existing data points in the training set and picks the k closest neighbors. The value of k chosen in this study is k = 3. In literature, the effectiveness of the kNN algorithm for EEG-based studies has already been established in a wide range of studies [68, 69].

#### 3.5.2 Adaptive boosting

Adaptive boosting, also known as AdaBoost, is a machine learning algorithm that is used for classification and regression tasks. It is an ensemble approach that concatenates multiple weak learners to construct a strong learner. The principal theory behind AdaBoost is to iteratively train a sequence of weak classifiers on diverse subsets of the training data. A weak classifier is a model that runs marginally better than a random guess, such as a decision tree with restricted depth. Each weak classifier is trained by assigning more weightage to the misclassified samples from the preceding classifiers. AdaBoost gives weights to each training sample during training, primarily setting them equally. After each weak classifier is trained, the weights of misclassified samples are raised, while the weights of accurately classified samples are reduced. This focuses the later weak classifiers on the samples that are challenging to classify correctly. In the final step, AdaBoost joins the weak classifiers by giving weights to them based on their performance. The classifiers with higher accuracy are given more weightage, and their predictions are merged to make the final prediction. The fused classifier is called the strong learner. AdaBoost classifier has been used in a wide range of EEG-based mental state classification studies especially depression [32, 70].

#### 3.5.3 Best-First Tree

The Best-First (BF) Tree is a machine-learning algorithm employed for decision tree induction. It is an expansion of the conventional decision tree algorithm, which fuses the best-first search approach with the concept of pruning to enhance efficacy and precision. In the BF-Tree algorithm, the best-first search strategy is utilized to determine the arrangement in which the attributes are assessed at each node of the decision tree. At each node, the algorithm decides on the attribute that gives the highest potential for information gain or drop in impurity, based on some valuation metric such as entropy or Gini index. The BF-Tree algorithm also encompasses pruning methods to thwart overfitting and improve the generalization ability of the decision tree. Pruning involves increasing the decision tree to its maximum size and then iteratively eliminating nodes that do not impact substantially the overall accuracy of the tree. This helps to simplify the tree structure and lower the risk of overfitting the training data. The BF-Tree algorithm continues the best-first search and pruning procedure up until a stopping criterion is met, such as reaching a certain depth or when further splitting does not provide significant improvement in the evaluation metric. BF-Tree has been used in a wide range of EEG-based mental state classification studies in the literature [71–73].

## 4 Experimental results

This section presents the experimental results for the proposed depression recognition framework using the EEG data recorded in resting state condition via a three-electrode recording system.

### 4.1 Subjects labeling

The dataset primarily contains data from individuals diagnosed with clinical depression and corresponding individuals with no depression, who serve as the healthy group. The choice of patients was done meticulously by expert psychiatrists in hospital settings to warrant precise diagnoses. Indoor and outdoor patients diagnosed with major depressive disorder (MDD) were collected from Lanzhou University Second Hospital in Gansu, China, based on recommendations from clinical psychiatrists. On the other hand, healthy participants were recruited using advertisements. The MDD patients were identified using a structured Mini-International Neuropsychiatric Interview (MINI) instrument that has been extensively used for the diagnosis of depression and has been established as an effective criterion for major depression diagnosis according to the Diagnostic and Statistical Manual of Mental Disorders [74]. Moreover, as a second filtering criterion to choose the patients of depression, Patient Health Questionnaire [75] was used to assess the depression level, and a scoring threshold of 5 was set for consideration as a patient going through depressive disorders. Moreover, even if a subject fulfills the criterion for being depressive, that subject was excluded from the study if going through a cerebral ailments or brain organ impairment and having a serious physical disease and severe suicidal inclinations. Moreover, the healthy subjects were also verified for any family history of mental disorders, and they were excluded from the study if any such condition occurs. Based on these criteria, EEG data of 55 subjects which include 26 subjects (15 males and 11 females belonging to the age bracket of 16–56-year-old) diagnosed with depression, as well as 29 healthy subjects (19 males and 10 females within the age brackets of 18–55-year-old) was recorded.

### 4.2 Feature selection

In the proposed study, the feature selection is achieved using the CFS method. The CFS method is applied to the extracted feature vector from each electrode (i.e., Fp1, Fpz, and Fp2) individually to obtain the selected subset of features. Table 1 presents the selected subset of features obtained from each EEG electrode. It can be observed from the table that for Fp1, the CFS algorithm selects 3 out of 12 features i.e., minimum amplitude, mean of absolute values of first and second difference. Similarly, for the *Fpz* electrode, the selected 4 features out of 12 include maximum and minimum amplitude and mean of absolute values of the first and second difference. Moreover, for the *Fp*2 electrode, the selected features include maximum amplitude, mean of absolute values of first and second difference, and average peak-to-peak signal value.

**Table 1:** Selected features for each individual EEG electrode obtained from correlation-based feature selection technique.

EEG Electrodes	Selected Subset of Features
Fp1	Minimum Amplitude.
	Mean of absolute values of first difference.
	Mean of absolute values of second difference.
Fpz	Maximum Amplitude.
	Minimum Amplitude.
	Mean of absolute values of first difference.
	Mean of absolute values of second difference.
Fp2	Maximum Amplitude.
	Mean of absolute values of first difference.
	Mean of absolute values of second difference.
	Mean Value.
	Peak to Peak Signal Value.

Next to this, a strategy is developed to select the common features based on the majority rule. i.e., the features appearing in the selected feature subset of at least two out of three electrodes. It can be observed from Table 1 that maximum and minimum amplitude and mean of absolute values of first and second difference are the features that are common in the selected feature subset of at least 2 out of 3 electrodes. Table 2 shows the common features obtained using the proposed strategy. The proposed feature selection strategy is compared to the early feature fusion (i.e., combining the features from all the electrodes and then performing feature selection to it) and late feature fusion (i.e., applying feature selection to features of all the electrodes individually and then fusing the selected features) schemes and superior results was achieved using the proposed feature selection mechanism.

**Table 2:** Common EEG features identified from the selected subset of features for each electrode.

Features	Mathematical Representations
Maximum Amplitude	$s_{e_{\text{max}}}=\text{max}\left\{s_{e}\left[n\right]\right\},$
Minimum Amplitude	$s_{e_{\text{min}}}=\text{min}\left\{s_{e}\left[n\right]\right\},$
Mean of absolute values of first difference	$MAD_{f_{e}}=\frac{1}{N}\sum \left|s_{e}\left[n+1\right]-s_{e}\left[n\right]\right|$
Mean of absolute values of second difference	$MAD_{s_{e}}=\frac{1}{N}\sum \left|s_{e}\left[n\right]-2*s_{e}\left[n-1\right]+s_{e}\left[n-2\right]\right|$

**Fig 4:**
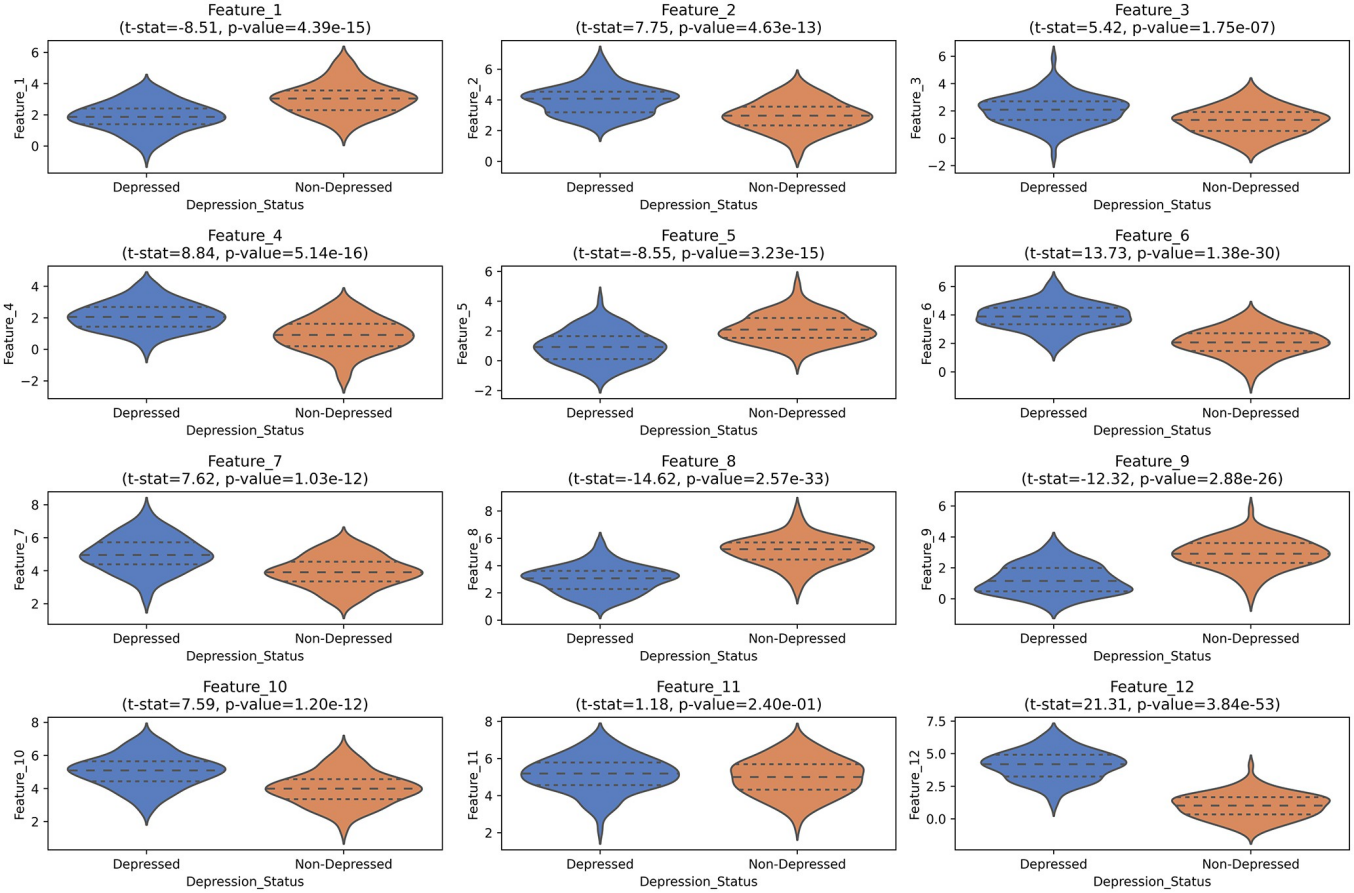
Volin plot for the selected common features obtained from the EEG data of the three electrodes i.e., Fp1, FpZ, Fp2.

Furthermore, the features which have been selected in the proposed depression screening framework using our feature selection strategy have been tested for statistical significance by applying t-test on each feature for depressed and non-depressed groups. The p-value obtained from the t-test results confirmed that 11 out of the selected 12 features are statistically significant having a p-value < 0.05. The violin plot for each feature shown in Fig 4 shows the statistical significance in the visual manner, confirming the results obtained from t-test.

### 4.3 Classification performance

Classification among depressed and healthy individuals is performed using three different classification algorithms which include BF-Tree, kNN, and AdaBoost. The classification results of the proposed scheme are computed using Weka 3.8 tool [76]. It is imperative to state that our suggested approach is subject agnostic, meaning the training and testing data are not from the same subject. The performance of the classification algorithms is evaluated based on metrics of accuracy, precision, recall, F-measure, and kappa statistics. Accuracy is a metric used to evaluate the rightness of a model’s predictions. It is expressed as the ratio of the number of correct predictions to the total number of predictions made by the model. Precision enumerates the proportionality of correctly predicted positive instances out of the total instances that the model projected as positive. Recall assesses the percentage of true positive predictions i.e., correctly classified positive instances out of all actual positive instances in the given data. F-measure is calculated as the harmonic mean of precision and recall. It merges precision and recalls into a single score. The F-measure goes from 0 to 1, where a value closer to 1 signifies reliable performance in terms of both precision and recall score. Kappa statistics also branded as Cohen’s Kappa is an evaluation parameter used to gauge the agreement among predicted and actual class labels while considering the agreement that could appear by chance alone.

Table 3 showcases the classification performance of the proposed depression classification framework using three different machine learning classifiers i.e., kNN, AdaBoost, and BF-Tree classifier. The results are evaluated in four varying feature combinations: no feature selection, early feature fusion-based feature selection, late feature fusion-based feature selection, and utilization of common features after feature selection. Classification using all features refers to a situation in which classifiers are applied to complete feature vector which in our case has a dimension of 220 x 36, where 220 are the number of instances and 36 are the number of features extracted from all the electrodes. This feature combination can be referred to as No Feature Selection. Secondly, Feature Selected with Early Feature Fusion means that first the features are extracted from each electrode and then they are combining into a single complete feature vector which in our study has a dimension of 220 x 36 and then feature selection is applied to this complete feature vector. The features selected after feature selection are subjected to classification. Thirdly, classification using Feature Selected with Late Feature Fusion means that the features are extracted from each electrode and feature selection is applied to each electrode to select the optimum subset of features. Then the selected features are fused/combined to construct a feature vector on which classification is performed. Common Features after Feature Selection refers to a setting in which first, the feature selection is performed on the extracted features from each electrode separately. After getting the selected features from each electrode, we choose the common set of features occurring in the majority i.e., 2 out of 3 electrodes and then those features are extracted from each electrode to construct a feature vector which is passed onto the classification stage.

**Table 3:** Performance comparison of the proposed depression classification scheme in terms of classifier used, FVL, accuracy, precision, recall, F-measure, and kappa statistics using 10-fold cross-validation.

Feature Combination	Classifier	FVL	Accuracy (%)	Precision	Recall	F-measure	Kappa
All Features	BF-Tree	36	81.81	0.81	0.81	0.81	0.63
	KNN	36	74.09	0.74	0.74	0.74	0.47
	Adaboost	36	78.63	0.79	0.78	0.78	0.56
Feature Selected with Early Feature Fusion	BF-Tree	8	94.09	0.94	0.94	0.94	0.88
	KNN	8	90.90	0.91	0.90	0.90	0.81
	Adaboost	8	76.81	0.77	0.76	0.76	0.53
Feature Selected with Late Feature Fusion	BF-Tree	12	92.72	0.92	0.92	0.92	0.85
	KNN	12	90.90	0.90	0.90	0.90	0.81
	Adaboost	12	76.81	0.77	0.76	0.76	0.53
Common Features after Feature Selection	BF-Tree	**12**	**96.36**	**0.96**	**0.96**	**0.96**	**0.92**
	KNN	12	91.36	0.91	0.91	0.91	0.82
	Adaboost	12	79.09	0.80	0.78	0.79	0.57

**Fig 5:**
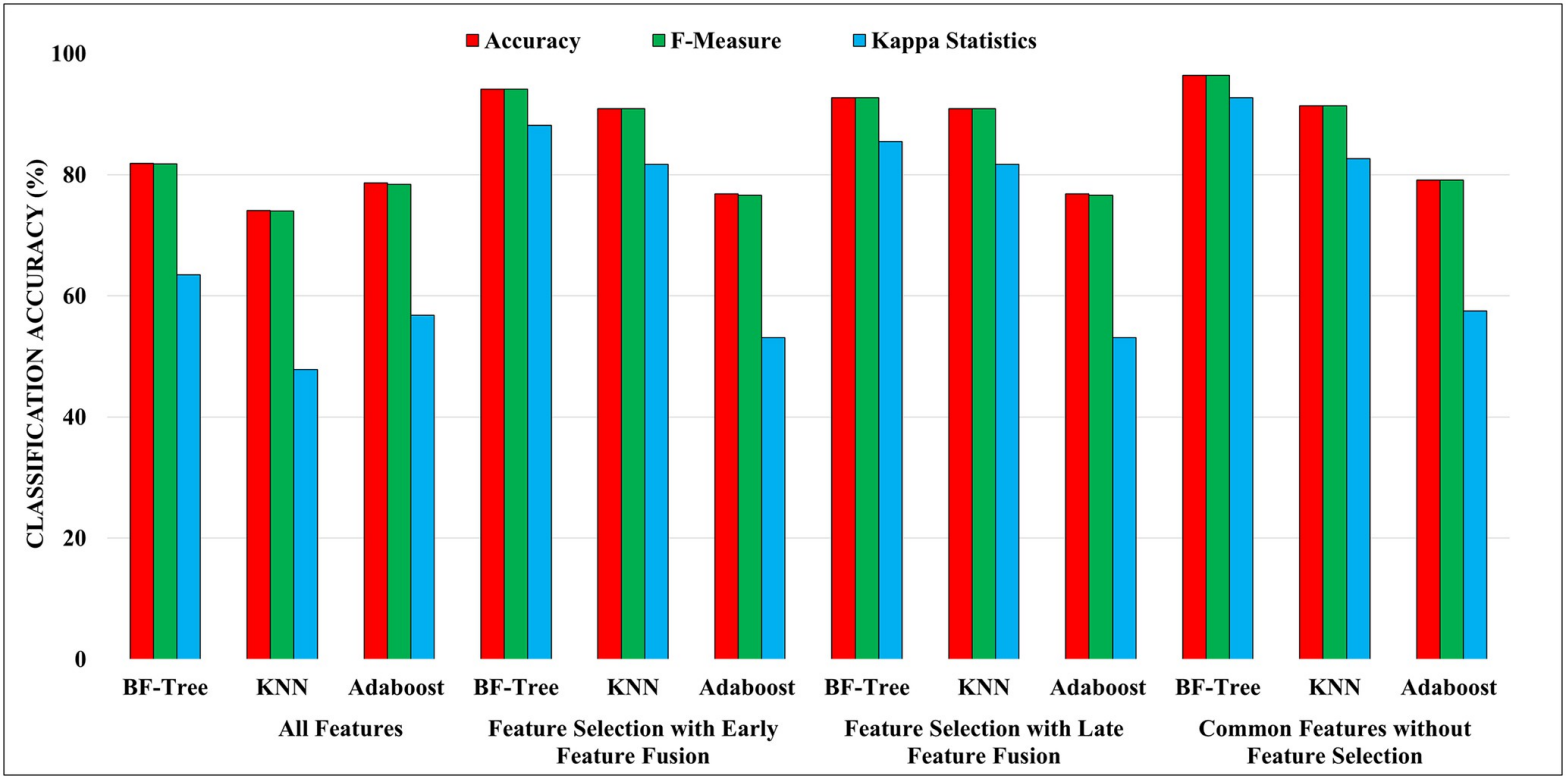
Performance comparison of the proposed depression classification scheme utilizing the classification accuracy, f-measure, and kappa statistics evaluation parameter.

It can be observed from the table that the best classification results are yielded when the common features obtained after applying feature selection to EEG data of each electrode are used with the BF-Tree classifier. An accuracy of 96.36% is achieved for mental depressive disorder vs healthy subjects classification using BF-Tree classifier using a feature vector length (FVL) of 12. Moreover, precision, recall, F-measure value of 0.964, and kappa statistics value of 0.92 are achieved for the proposed scheme. Moreover, we have incorporated a nested cross-validation scheme to ensure robust model evaluation and avoid potential biases. Table 4 presents the results of the proposed scheme using nested cross-validation scheme. It can be observed that a highest classification accuracy of 97.04% is achieved using BF-Tree classifier, which is comparable to the results obtained from the aggregated results of each cross-validation fold. These results using nested cross-validation will enhance the validity and reliability of our proposed scheme. Fig 5 presents the graphical representation of the classification performance of the proposed mental depressive disorder scheme in terms of classification accuracy, F-measure, and kappa statistics evaluation parameters. It can be observed from the figure that the highest classification accuracy is attained by the BF-tree classifier when common features obtained after feature selection are utilized.

**Table 4:** Performance comparison of the proposed depression classification scheme in terms of classifier used, feature vector length (FVL), accuracy, precision, recall, F-measure, and kappa statistics using Nested cross-validation.

Feature Combination	Classifier	FVL	Accuracy (%)	Precision	Recall	F-measure	Kappa
All Features	BF-Tree	36	81.36	0.84	0.81	0.81	0.63
	KNN	36	56.82	0.57	0.57	0.54	0.12
	Adaboost	36	75.91	0.79	0.70	0.73	0.51
Feature Selected with Early Feature Fusion	BF-Tree	8	94.27	0.98	0.97	0.97	0.9
	KNN	8	93.31	0.99	0.99	0.99	0.98
	Adaboost	8	77.27	0.80	0.76	0.76	0.55
Feature Selected with Late Feature Fusion	BF-Tree	12	95.45	0.96	0.95	0.95	0.91
	KNN	12	94.79	0.99	0.99	0.99	0.98
	Adaboost	12	77.27	0.80	0.74	0.76	0.55
Common Features after Feature Selection	BF-Tree	**12**	**97.04**	**0.98**	**0.97**	**0.97**	**0.93**
	KNN	12	95.22	0.99	0.99	0.99	0.98
	Adaboost	12	79.09	0.82	0.77	0.78	0.58

**Fig 6:**
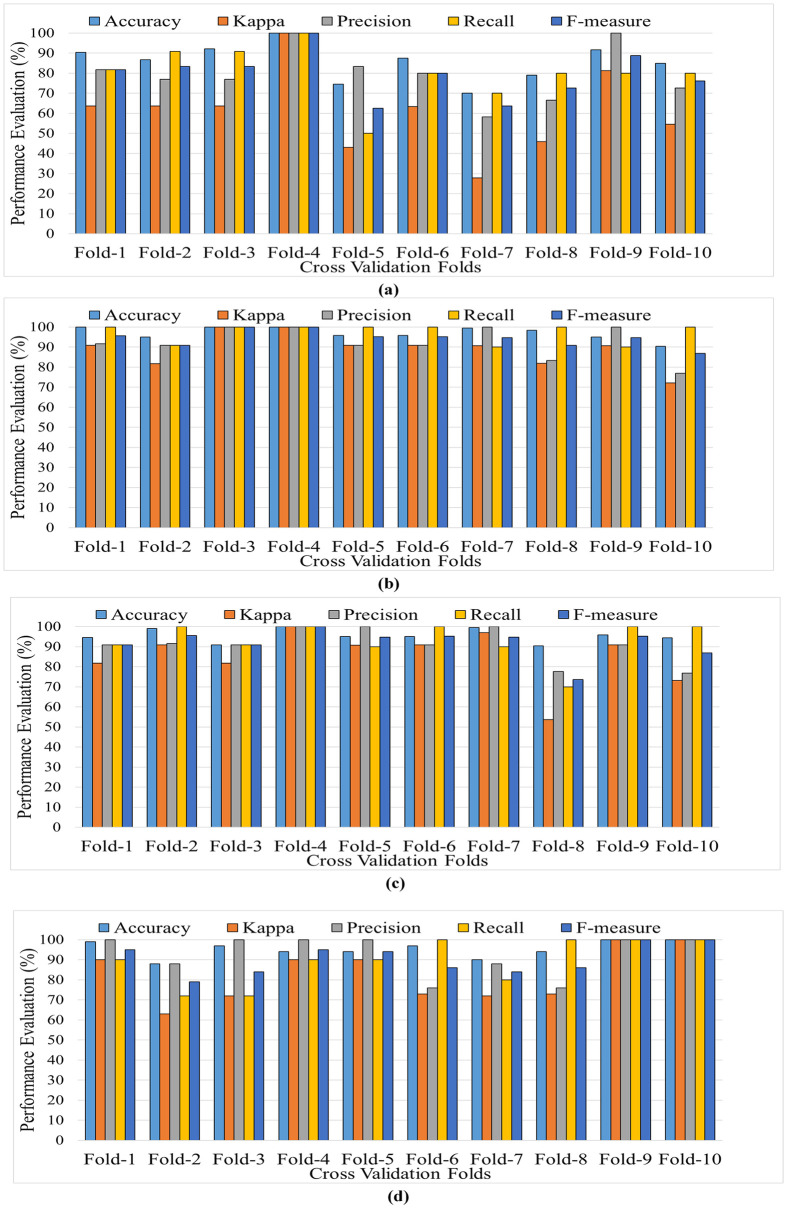
The graphs of the BF-Tree classification model using 10-fold cross-validation for the EEG signals recorded in the rest-state for (a) without feature selection (b) feature selection with early feature fusion (c) feature selection with late feature fusion (d) common features after feature selection.

The classification results are computed using a 10-fold cross-validation method. This approach requires arbitrarily partitioning the data into k equal segments. During the training stage, k-1 segments are utilized, while the remaining segment is retained for testing. This process is repeated until all segments have been assessed in the testing phase. For this study, a value of *k* = 10 was chosen, resulting in the random division of the data into 10 equal sections. Fig 6 depicts the performance values obtained via a 10-fold cross-validation test, demonstrating the efficacy of the proposed depression classification scheme.

**Fig 7:**
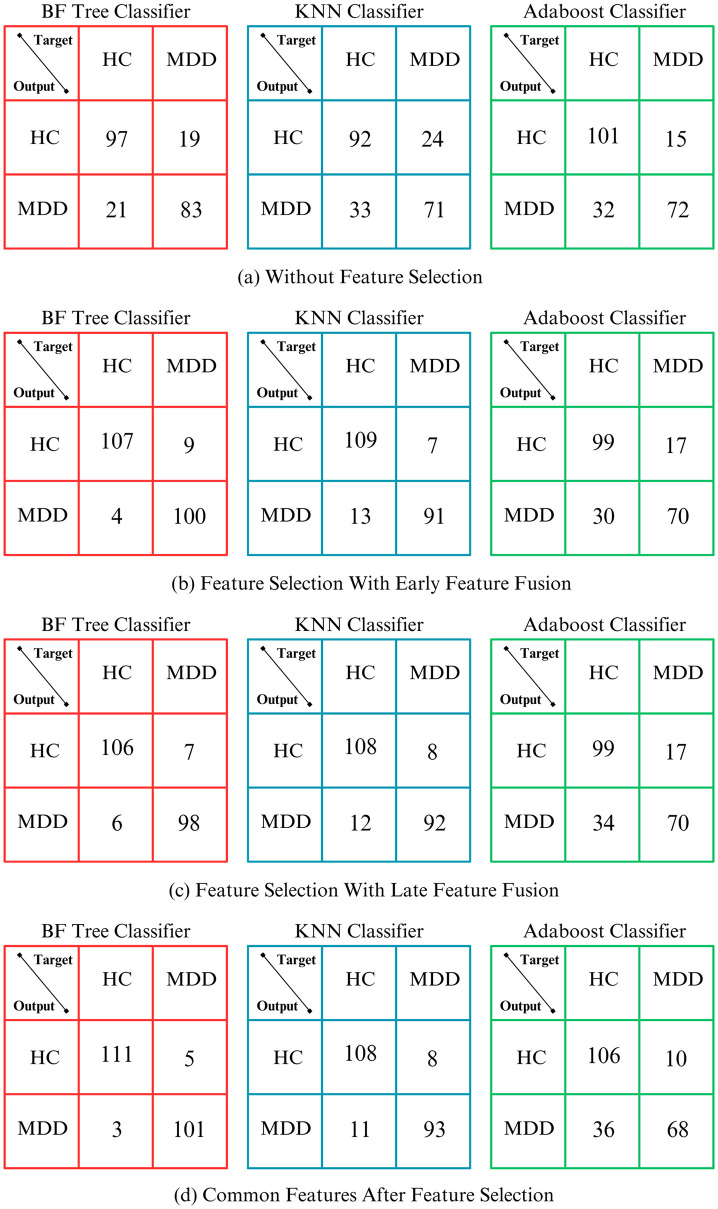
Confusion Matrices for BF-Tree, KNN, and AdaBoost classifier for (a) without feature selection (b) feature selection with early feature fusion (c) feature selection with late feature fusion (d) common features after feature selection.

The confusion matrix is a graphical demonstration of the performance of a classification model, presenting a table that confirms the number of true positives, true negatives, false positives, and false negatives. The confusion matrix allows for the calculation of diverse sets of evaluation metrics such as accuracy, precision, recall (sensitivity), specificity, and F-measure. It provides insights into the model’s ability to correctly classify instances and helps in understanding the types of errors yielded by the model. Fig 7 presents the confusion matrices for the three classifiers i.e., BF-Tree, KNN, and AdaBoost classifier. The results are shown for all four feature combinations as stated in Table 3. It can be observed from the confusion matrices that BF-Tree realizes better results as compared to KNN and AdaBoost classifier for all the feature combinations. However, the proposed common feature extraction after the feature selection approach yields the best classification results for all the classifiers among all the feature combinations techniques. Moreover, BF-Tree has the lowest number of misclassifications for healthy and mental depressive disorder patients. For the healthy controls, out of 116 instances, 111 were correctly classified and only 5 instances got misclassified. Similarly, for the patients diagnosed with mental depressive disorder, 101 out of 104 instances were correctly classified, thus resulting in only 3 misclassifications thus establishing the effectiveness of the proposed mental depressive disorder scheme.

**Fig 8:**
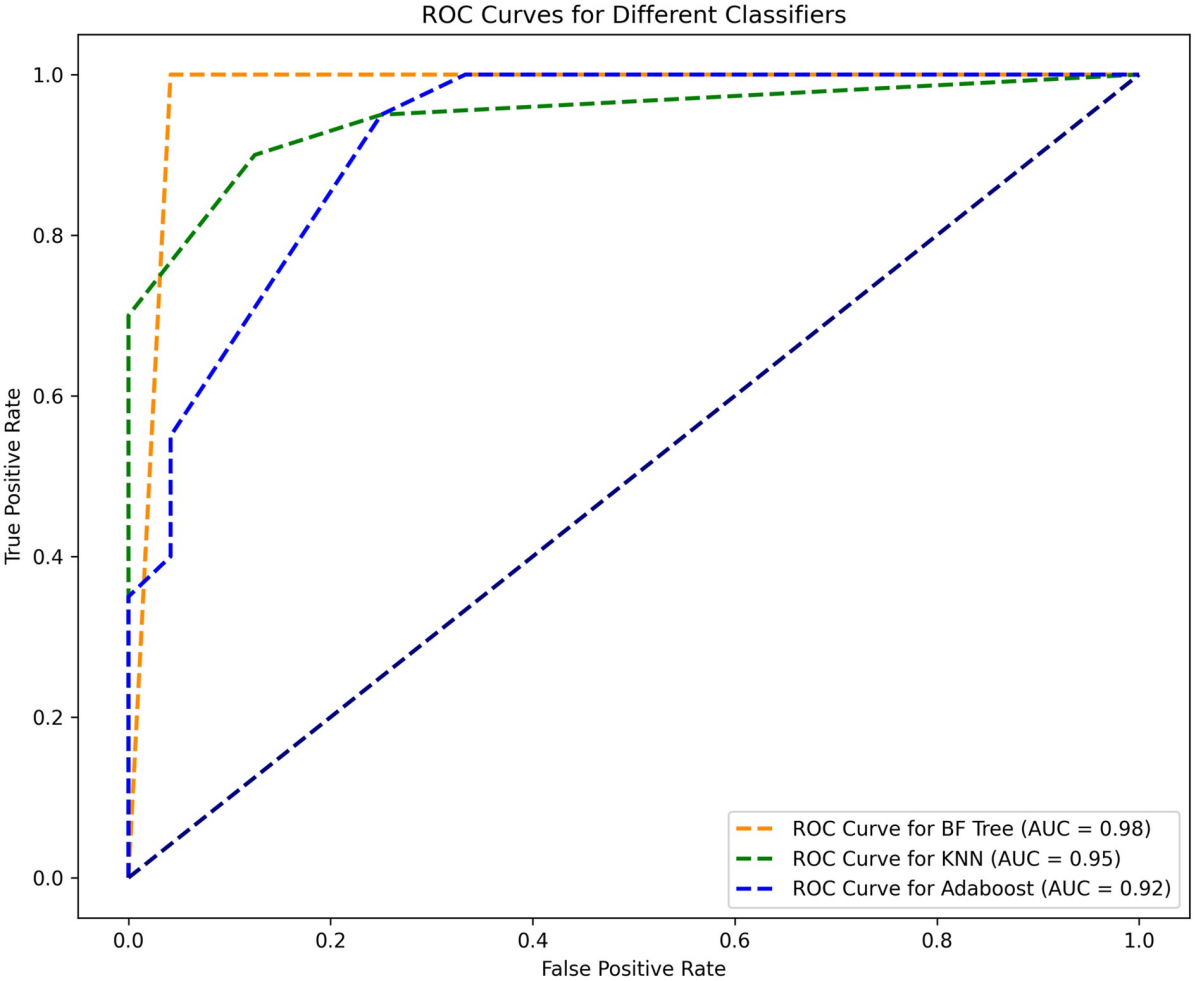
Receiver operating characteristic for BF-Tree, KNN, and AdaBoost classifier for the proposed depression screening framework.

The Receiver Operating Characteristic (ROC) Curve is a graphical representation of the performance of a binary classification model across different discrimination thresholds. It plots the true positive rate (sensitivity) against the false positive rate (1—specificity) for various threshold values. The ROC curve typically rises steeply at the beginning, indicating that at lower thresholds, the true positive rate increases while the false positive rate is also low. As we move toward the right, the trade-off between sensitivity and specificity becomes more pronounced. Ideally, we want the ROC curve to be as close as possible to the top-left corner of the plot. This signifies high sensitivity and low false positive rate. Moreover, a higher area under the ROC curve (AUC-ROC) generally indicates better model performance. The area under the ROC curve is a single value that summarizes the overall performance of the model across all possible thresholds. A model with an AUC-ROC of 1.0 is perfect, while a model with an AUC-ROC of 0.5 performs no better than random chance. Fig 8 presents the ROC curve of the BF-Tree, KNN, and AdaBoost classifier for our proposed depression screen framework. It can be observed from the ROC curve that BF-Tree classifier has the highest true positive and the lowest false positive rate as compared to KNN and AdaBoost classifier, depicting the effectiveness of the proposed scheme. Moreover, a comparison of the area under the curve (AUC) for all the classifiers depicts the highest value of AUC of 0.98 for the BF-Tree classifier as compared to KNN and AdaBoost models.

## 5 Comparison and discussion

The proposed study presents a mental depressive disorder detection scheme using EEG data recorded in the resting-state condition. This section presents the comparative analysis of the proposed scheme with the state-of-the-art methods available in the recent literature. Table 5 illustrates a comparative examination of the proposed approach and the currently leading depression classification approaches that utilize EEG signals. The studies chosen for the purpose of comparison includes [1, 29, 30, 32, 37–39, 56, 77, 78]. These mental depressive disorder classification studies can be alienated into two categories i.e., mental depressive disorder detection based on MODMA dataset [1, 38, 39, 56, 78] or other public or private datasets [29, 30, 32, 37, 77]. Our proposed scheme utilized the MODMA dataset recorded using a 3 electrodes headset best suited for pervasive applications in the resting-state condition hence for a fair comparison we have chosen a significant number of studies that utilized the MODMA dataset. The comparison of the proposed scheme with the available mental depression detection schemes are conducted in terms of classifiers used, number of participants, number of EEG electrodes, feature vector length, and the accuracy achieved.

**Table 5:** Performance evaluation of the anticipated scheme with state-of-the-art approaches for human mental depressive disorder classification using EEG.

Ref, Year	Classifier	Dataset	No. of Subjects	No. of Electrodes	FVL	Accuracy
[56], 2023	*CNN*	MODMA	53	128	10	97%
[78], 2023	*KNN*	MODMA	13	13	256	83.96%
[37], 2023	*SVM*	MPHC dataset	49	19	1176	94.03%
[32], 2022	*KNN*	Private Dataset	20	18	162	95%
[38], 2022	*CNN*	MODMA	53	128	-	89.63%
[39], 2022	*CNN-GRU*	MODMA	53	128	12	99.33%
[29], 2021	*KNN*	Private Dataset	44	2	13	98.79%
[30], 2020	*CBEM*	Private Dataset	34	16	304	92.65%
[1], 2020	*KNN*	MODMA	43	3	12	72.25%
**Proposed (10-fold), 2023**	***BF-Tree***	MPHC	**49**	**3**	12	**90.15%**
**Proposed (Nested CV), 2023**	***BF-Tree***	MPHC	**49**	**3**	12	**88.65%**
**Proposed (10-fold), 2023**	***BF-Tree***	MODMA	**55**	**3**	12	**96.36%**
**Proposed (Nested CV), 2023**	***BF-Tree***	MODMA	**55**	**3**	12	**97.04%**

* CV: Cross-Validation

Comparing the number of participants for the studies which utilize the MODMA dataset to our proposed scheme, our study has 55 participants whereas, the maximum number of participants in MODMA-based studies in the literature is 53. However, the depression detection studies conducted on other public [37, 77] and private [30] datasets have 49, 12, and 70 subjects respectively. The major limitation of these datasets with a higher number of subjects are not publicly available for future research. Furthermore, analyzing the number of electrodes utilized for depression detection, it can be observed that our proposed scheme utilizes EEG data from only 3 electrodes, whereas all other MODMA dataset-based studies have utilized a larger number of EEG electrodes except [1], which involves EEG data of 3 electrodes. However, it can be observed that our proposed scheme achieves a higher depression classification accuracy of 96.36% as compared to the study conducted in [1] which has a classification accuracy of 72.25%. Moreover, for all the other depression detection studies conducted on the MODMA dataset, the highest classification accuracy of 99.32% is achieved using EEG data from 128 electrodes, which in contrast to our proposed scheme is significantly computationally intensive with only a slight increase in depression detection accuracy. Furthermore, the highest depression classification accuracy of 98.79% is achieved using a private EEG dataset comprising of 44 subjects and 2 electrodes in a study conducted in [29].

In terms of feature vector length, our proposed scheme employed only 12 features from 3 EEG electrodes whereas all other earlier studies have used feature vector length greater than 12 except the studies conducted in [1, 38, 56]. It could be observed from the results of the study in [1], that even though the feature vector length used for classification was 12 with 3 electrodes but the classification accuracy achieved was only 72.25% which is significantly lower than our proposed methodology. This is because the features used in the proposed depression screening method are statistically significant for depression identification as indicated by the p-values of the t-test and Violin plot presented in Fig 4. Therefore, these features yield a better depression classification performance as compared to the earlier studies. Moreover, another study discussed in [38] achieved a depression classification accuracy of 99.33% by utilizing a feature vector length of 12 with 128 EEG electrodes. The classification accuracy of this study is higher as compared to our proposed scheme but with a huge number of EEG electrodes thus significantly increasing the computational complexity of the scheme whereas our proposed system was able to achieve an accuracy of 96.36% with only 3 EEG electrodes. Furthermore, another study expounded in [56] developed a feature vector of 10 by utilizing the EEG data from 128 electrodes and achieving a depression classification accuracy of 97% which is slightly above the accuracy of our proposed scheme but with a significantly higher number of EEG electrodes.

We have evaluated the features of our proposed scheme on different datasets mentioned in Table 5 to assess the generalizability of our approach. There are three different datasets used in the studies mentioned in Table 5 of the manuscript, which include MODMA, MPHC and Private dataset. MODMA is the same dataset which we have used in our study, and the results obtained for our scheme are highest among all the MODMA dataset-based studies mentioned in the Table in terms of number of electrodes, feature vector length and number of subjects. Moreover, we could not obtain the private dataset mentioned in [29, 30, 32] to evaluate the features of our scheme on it. However, we assessed the generalizability of our extracted features on the MPHC dataset. Evaluating the features derived from our proposed scheme on the MPHC dataset, an accuracy of 90.15% and 88.65% is achieved using BF-Tree classifier with a feature vector length of 12 using only 3 electrodes using 10-fold and nested cross-validation, respectively, whereas in comparison, the study mentioned in [37] achieved an accuracy of 94.03% using 19 electrodes and a feature vector length of 1176 features. Our features produced comparable classification accuracy by using a smaller number of electrodes and feature vector length, advocating the fact that the extracted features can be used for computing the generalizable depression classification results. This additional experimentation enhanced the validity of our results and provided a more comprehensive understanding of the method’s performance across diverse scenarios.

Hence it can be clinched that our proposed depression classification framework was able to discriminate the mental depressive disorder patients from the healthy controls with an accuracy of 96.36% using EEG data from only 3 electrodes and a small feature vector length of 12, thus dropping the computational cost of the system as equated to the existing depression classification studies offered in the literature which were able to realize the comparable accuracy with a substantial amount of computational burden. Furthermore, the results of the proposed mental depressive disorder classification scheme were computed on a publicly available MODMA dataset which was logged in the clinical settings under the direction of medical experts thus adding to the legitimacy of the EEG recording and making it easier for the future researcher to replicate the existing results.

Our study utilized a publicly available dataset that predominantly focused on the frontal lobe area of the brain. While we acknowledge the importance of considering the influence of all brain lobes for a comprehensive understanding, our rationale for the dataset selection was driven by specific research objective of enhancing the classification accuracy of the depression classification scheme while keeping the number of EEG electrodes minimum. The frontal lobe of the human brain, being implicated in various cognitive functions and often associated with complex behaviors, served as a primary area of interest for our investigation. By concentrating on this specific region, we aimed to provide a detailed exploration of its significance within the scope of our research questions. However, we acknowledge the limitation posed by the exclusion of information from other lobes. To address this concern, detailed analyses encompassing a broader range of brain areas in future research endeavors need to be performed. This approach will facilitate a more holistic understanding of the brain’s functional dynamics and its potential impact on our findings. It is important to note that while our dataset choice may introduce a certain degree of bias toward the frontal lobe, we believe that our focused examination of this region contributes valuable insights to the existing literature regarding depression classification. Nevertheless, we recognize the necessity of extending our investigations to incorporate a more diverse dataset in subsequent studies to offer a more comprehensive perspective on brain functioning.

## 6 Conclusion

The rise in the use of ubiquitous devices facilitates us to exploit EEG wearable headsets for recognizing depression. This paper presents a mental depression detection framework using EEG recording of 55 subjects in the resting-state condition using three frontal-lobe electrodes i.e., *Fp*1, *Fp*2, and *Fpz*. The selected set of 4 features include minimum and maximum amplitude and mean of absolute values of first and second difference are obtained from each of the EEG electrodes. The highest classification accuracy of 96.36% is achieved using the BF-Tree classifier with a feature vector length of 12, which is considerable in comparison to techniques existing already in the literature. However, in the future, frequency domain features extracted from the recorded EEG data can be involved to get a more comprehensive understanding of the neural patterns occurring in the human brain. Nevertheless, by developing extensive datasets sourced from diverse ethnic backgrounds, it is likely to produce more powerful models. These models could then be employed to recognize depression and other neurological disarrays at an early stage by investigating EEG signals.
